# Balancing benefits and risks in lung cancer therapies: patient preferences for lung cancer treatment alternatives

**DOI:** 10.3389/fpsyg.2023.1062830

**Published:** 2023-06-21

**Authors:** Serena Oliveri, Lucilla Lanzoni, Jorien Veldwijk, G. Ardine de Wit, Serena Petrocchi, Rosanne Janssens, Elise Schoefs, Meredith Y. Smith, Ian Smith, Kristiaan Nackaerts, Marie Vandevelde, Evelyne Louis, Herbert Decaluwé, Paul De Leyn, Hanne Declerck, Francesco Petrella, Monica Casiraghi, Giulia Galli, Marina Chiara Garassino, Charis Girvalaki, Isabelle Huys, Gabriella Pravettoni

**Affiliations:** ^1^Applied Research Division for Cognitive and Psychological Science, IEO, European Institute of Oncology IRCCS, Milan, Italy; ^2^Erasmus School of Health Policy and Management and Erasmus Choice Modelling Centre, Erasmus University Rotterdam, Rotterdam, Netherlands; ^3^Julius Center for Health Sciences and Primary Care, University Medical Center Utrecht, Utrecht University, Utrecht, Netherlands; ^4^Department of Pharmaceutical and Pharmacological Sciences, KU Leuven, Leuven, Belgium; ^5^Department of Risk Management, Alexion Pharmaceuticals, Inc., Boston, MA, United States; ^6^Department of Regulatory and Quality Sciences, School of Pharmacy, University of Southern California, Los Angeles, CA, United States; ^7^Department of Respiratory Oncology, University Hospital Leuven, Leuven, Belgium; ^8^Department of Thoracic Surgery, KU Leuven, Leuven, Belgium; ^9^Department of Thoracic Surgery, IEO, European Institute of Oncology IRCCS, Milan, Italy; ^10^Department of Oncology and Hemato-oncology, University of Milan, Milan, Italy; ^11^Department of Medical Oncology, Fondazione IRCCS Istituto Nazionale dei Tumori, Milan, Italy; ^12^EUA Affairs Manager, European Cancer Patient Coalition, Brussels, Belgium

**Keywords:** non-small cell lung cancer, patient preference, discrete-choice experiment, mixed logit model, maximum acceptable risk, minimum acceptable benefit

## Abstract

**Background:**

In the treatment of Non-Small Cell Lung Cancer (NSCLC) the combination of Immuno- Oncotherapy (IO) and chemotherapy (CT) has been found to be superior to IO or CT alone for patients’ survival. Patients and clinicians are confronted with a preference sensitive choice between a more aggressive treatment with a greater negative effect on quality of life versus alternatives that are less effective but have fewer side effects.

**Objectives:**

The aims of this study were to: (a) quantify patients’ preferences for relevant attributes related to Immuno-Oncotherapy treatment alternatives, and (b) evaluate the maximum acceptable risk (MAR)/Minimum acceptable benefit (MAB) that patients would accept for treatment alternatives.

**Methods:**

An online preference survey using discrete-choice experiment (DCE) was completed by NSCLC patients from two hospitals in Italy and Belgium. The survey asked patients’ preferences for five patient- relevant treatment attributes. The DCE was developed using a Bayesian D-efficient design. DCE analyses were performed using mixed logit models. Information regarding patient demographics, health literacy, locus of control, and quality of life was also collected.

**Results:**

307 patients (158 Italian, 149 Belgian), stage I to IV, completed the survey. Patients preferred treatments with a higher 5-year survival chance as the most important attribute over all the other attributes. Preference heterogeneity for the attribute weights depended on health literacy, patients’ age and locus of control. Patients were willing to accept a substantially increased risks of developing side effects in exchange for the slightest increase (1%) in the chance of surviving at least 5 years from the diagnosis of cancer. Similarly, patients were willing to accept a switch in the mode of administration or complete loss of hair to obtain an increase in survival.

**Conclusion:**

In this study, the proportion of respondents who systematically preferred survival over all other treatment attributes was particularly high. Age, objective health literacy and locus of control accounted for heterogeneity in patients’ preferences. Evidence on how NSCLC patients trade between survival and other NSCLC attributes can support regulators and other stakeholders on assessing clinical trial evidence and protocols, based on patients’ conditions and socio-demographic parameters.

## 1. Introduction

Non-small cell lung cancer (NSCLC) is the most prevalent type of lung cancer accounting for 80–90% of total cases (Duma et al., 2019). Due to the mild nature and non-specificity of early-symptoms, NSCLC is typically not diagnosed until late stages of the disease when surgical resection of the tumor is no longer an option (Ironmonger et al., 2015; Kocher et al., 2015; Siegel et al., 2020). This late diagnosis often results in a poor prognosis of NSCLC patients, with over 50% of the patients dying within a year after diagnosis and only 20.5% surviving 5 years post-diagnosis (Howlader et al., 2017). For several decades, the standard frontline treatment for advanced stages of NSCLC has consisted of platinum-based chemotherapy (Zappa and Mousa, 2016; Hanna et al., 2020), but recent breakthrough advancements in immunotherapy with immune checkpoint inhibitors have substantially changed the landscape of cancer care. Immunotherapy has been found to significantly improve the 5-year survival rate compared to platinum-based chemotherapy (Reck et al., 2016) and combination chemo-immunotherapy has proven superior to chemotherapy alone (Langer et al., 2016; Gandhi et al., 2018).

However, treatments for NSCLC differ widely in terms of benefits, side effects and mode of administration. In exchange for a higher response rate and a longer duration of response compared to standard chemotherapy, chemo-immunotherapy is associated with toxicity profiles which are significantly different than immunotherapy or chemotherapy alone (Shafique and Tanvetyanon, 2019). Side-effects can include severe fatigue, nausea, vomiting, paraesthesia, and anemia in the acute phase, with persistent infertility and neurotoxicity at later stages as well as damage to the skin, lungs, gastrointestinal tract, liver, endocrine glands, and skeletal muscle (Naidoo et al., 2015; Cousin and Italiano, 2016). Immunotherapy alone is generally better tolerated than combination therapy, with milder side effects such as skin rash and itch, mild diarrhea, fatigue, and subclinical thyroid dysfunction (Lee Ventola, 2017; Brahmer et al., 2018). Further, practical aspects of the treatment are different with chemo-immunotherapy administered via intravenous infusions lasting 4–5 h, while immunotherapy infusions typically last <1 h. Due to the lack of a clear best choice between these alternatives, decisions concerning NSCLC treatment can be considered “preference sensitive” whereby the “best” treatment depends on how the patient values the trade-offs between more aggressive options with greater impact on the quality of life versus alternatives that may be less taxing but also less effective.

The aim of the current study is to (a) quantify NSCLC patient preferences for different benefits and risks of immunotherapy, (b) identify how much risk an individual is willing to accept in exchange for a given degree of benefit (i.e., maximum acceptable risk) and how much benefit an individual requires in order to offset a given risk (i.e., minimum required benefit), and (c) investigate what factors (including psychological factors such as health literacy and health locus of control) can explain preference heterogeneity.

## 2. Materials and methods

### 2.1. Participants

Participants were selected and referred to the PREFER research team by the treating oncologists at the Thoracic Oncology Division of the European Institute of Oncology and the National Cancer Institute in Milan, and at the Respiratory Oncology Department of the KU Leuven University Hospital in Leuven. Patients were eligible if they were stage I to IV NSCLC patients, over 18 years of age, and able to read and speak Italian or Dutch (Monzani et al., 2021). Patients were approached via phone or in the hospital waiting room before a visit, and if they agreed to participate, they received the information sheet and a consent form by email. The study was approved by the Ethical Committee of the European Institute of Oncology IRCCS (IEO, Milan, Italy; reference R1142/20-IEO 1206) and the “Ethische Commissie Onderzoek UZ/KU Leuven” (Belgium; reference S63007).

### 2.2. Methodology of discrete choice experiment

A discrete choice experiment (DCE) was used to quantify patient preferences for different treatment regimens (Monzani et al., 2021). DCEs are based on the random utility theory (RUT), which assumes that the value (utility) of a product is determined by the value of the individual characteristics that define the product (i.e., attributes; McFadden, 1973). In a DCE, the respondent is presented with a series of choice tasks consisting of two or more treatment alternatives to choose from. These alternatives are defined by varying combinations of different levels of treatment characteristics (or attributes; Ryan et al., 2008). Each respondent completes a pre-determined number of choice tasks from which the individual attribute utility can be estimated by modelling how different attributes (and their levels) were associated with the respondent choices across the tasks (Viney et al., 2005). In our DCE, participants were presented with choice tasks consisting of two hypothetical treatment alternatives described using five attributes with three levels per attribute (Raut et al., 2020). These attributes and levels were identified through focus group discussions with NSCLC patients in Italy and Belgium, and ranked by patients through the Nominal Group Technique. The first five ranked attributes were selected for the DCE and further refined through discussions with clinicians and patient representatives (Petrocchi et al., 2021). The attributes and levels used in the DCE are listed in Table 1 (for a more detailed description of attributes and their definitions see Monzani et al. (2021).

**Table 1:** Exact wording of the attributes and levels used in the DCE.

Attribute	Definition	Levels
How the treatment is given to you	How the cancer treatment is given to you and the length of time each treatment takes.	This can either be: Infusion (Injection administered into your veins) that requires a hospital stay of 1 day (about 24 h) Infusion (Injection administered into your veins) that requires a hospital stay of half a day (about 12 h); Oral treatment (by swallowing), and no hospital stay is required.
Chance of surviving 5 years after starting this cancer treatment	The chance of still being alive 5 years after starting this cancer treatment.	This chance can either be: 10% -meaning that 10 people out of 100 people that started the treatment are still alive after 5 years, and 90 people died within those 5 years 20% -meaning that 20 people out of 100 people that started the treatment are still alive after 5 years, and 80 people died within those 5 years 40% -meaning that 40 people out of 100 people that started the treatment are still alive after 5 years, and 60 people died within those 5 years
Chance of long lasting skin problems	The chance that skin problems occur after treatment. This skin problem lasts at least a month and could be a rash, severe itching, bleeding and/or dryness	This chance can either be: 10% (10 out of 100)a 20%(20 out of 100)a 40% (40 out of 100)a
Chance of being extremely tired	This refers to feeling completely exhausted and lacking energy even after limited activities. It lasts as long as the treatment takes to be administered.	This chance can either be: 10% (10 out of 100)a 40% (40 out of 100)a 60% or (60 out of 100)a
Severity of hair loss	The type and amount of hair loss. It lasts as long as the treatment takes to be administered.	This can either be: no hair loss weakening/thinning of hair complete loss of hair

**Figure 1:**
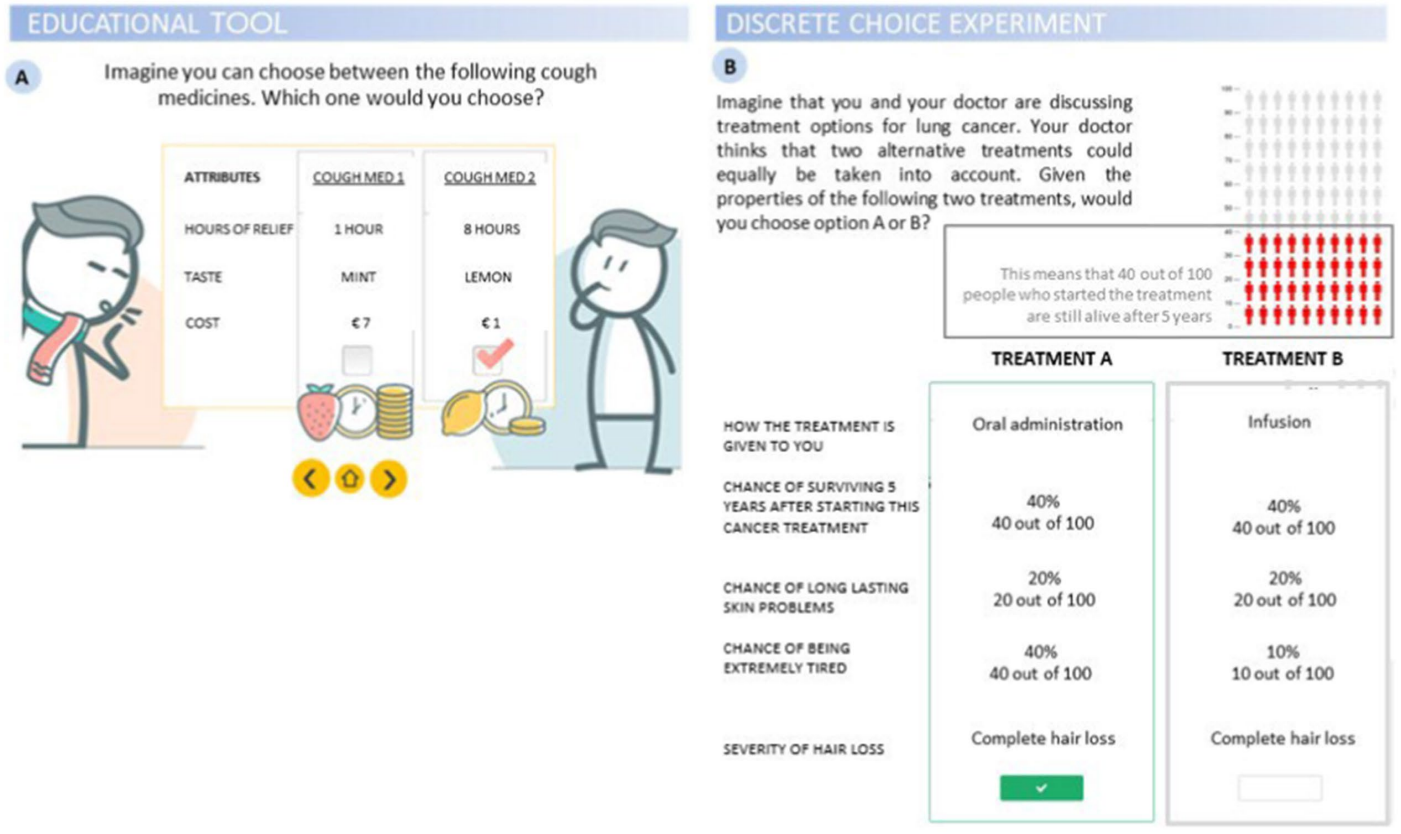
Illustrations from the survey. **(A)** Educational tool introducing discrete choice experiment (DCE) with an imaginary choice for a cough medicine; **(B)** DCE task probing participants to choose between two alternative treatment options given a set of attributes, with an example of the graphical representation of probabilities from one of the 5 attributes.

A Bayesian D-efficient design consisting of two alternative hypothetical treatments was constructed for the DCE using Ngene (ChoiceMetrics. Sydney, Australia). The 36 unique choice tasks generated were divided over three blocks so each respondent answered 12 choice tasks (respondents were randomized to either of the blocks). The survey was pilot tested in think-aloud interviews in Italy (*N* = 5) and the outcomes of a conditional logit model were used to improve the final experimental design. Interactions between the attributes ‘Chance of surviving 5 years after starting this cancer treatment’ and, respectively, ‘Chance of long-lasting skin problems’, ‘Chance of being extremely tired’ and ‘How the treatment is given to you’ were accounted for in this design. An example of a DCE choice task can be found in Figure 1B.

### 2.3. Survey

Data collection took place remotely through individualized links generated by Sawtooth software offered via a server at Uppsala University, Sweden. Survey questions were originally developed in English and translated into Italian and Dutch by a professional translation service.

Prior to completing the survey, respondents read the information sheet and gave their informed consent by selecting the relevant box. The survey started with questions on patients’ demographics (i.e., country of residence, age, gender, level of education, family and relationship status, family history of cancer) and medical background (i.e., cancer stage, type of treatment and lines of treatment). Patients enrolled might have undergone more than one treatment at the time of enrolment for this study, thus a multiple-choice question for “type of treatment” was applied. This was followed by an educational video which introduced health-related terminology, explaining the basic aims of the choice tasks, and instructing participants as to how to complete the choice task (Figure 1A). Respondents then answered the choice tasks in the DCE.

Then respondents completed measures assessing psychological constructs that have previously been found to explain heterogeneity in preferences for health-related decisions (Russo et al., 2019). The first construct was health literacy, which refers to the patient’s ability to read, understand, and use healthcare information appropriately (Kindig et al., 2004; Berkman et al., 2010; Sørensen et al., 2012). Higher health literacy is associated with higher engagement in medical decision-making (Goggins et al., 2014) and reduced reliance on physicians as the main source of health information (Gaglio et al., 2012). This was measured using Chew’s Set of Brief Screening Questions and the Newest Vital Sign (NVS). Chew’s Set of Brief Screening Questions (Chew et al., 2004) is a self-reported, validated measure of health literacy which includes three questions probing the patient’s need for external help in processing health-related material, confidence in filling out medical forms, and difficulty learning about their medical condition. Objective health literacy was measured with the NVS (Weiss et al., 2005), which probes patients to answer six questions based on the information contained in a mock ice cream nutrition label.

Next, health locus of control (LoC) was measured. LoC reflects a tendency to attribute health outcomes to either one’s own responsibility or to external forces (Wallston et al., 1978). LoC was found to be a better predictor of information seeking behavior and involvement in medical decision-making than such demographic variables as age, sex and educational level (Snell et al., 1991; Braman and Gomez, 2004; Rowe et al., 2005; Schneider et al., 2006). LoC was measured using the Multidimensional Health Locus of Control – Form C (MHLC – C; Wallston et al., 1994) consisting of four subscales: Internal, Chance, Doctors, and Others, assessing the extent to which respondents believe the given factors affect their health status or progress of their disease.

Then respondent health related quality of life (HRQoL) was assessed using the EQ-5D [EQ-5D-5L (EuroQol Research Foundation, 2019)]. The EQ-5D measures HRQoL I five dimensions: mobility, self-care, daily activities, pain/discomfort, and anxiety/depression. Finally, 5-point Likert scale questions assessed patients’ acceptability of the educational tool, DCE and length of the survey (“How easy or difficult was it for you to understand the questions?”; “How easy or difficult was it for you to answer the questions?”; “Did the educational material (i.e., the video instruction) help you understand the questions?”; “What did you think about the length of the questionnaire?”).

We pre-tested the survey with five patients in Italy, who completed a draft version of the survey at home on their personal computer or tablet, while communicating with the researcher via video-conferencing tools. Patients were instructed to verbalize any difficulty they encountered in completing the survey, including doubts, or obstacles they experienced in each section (Oliveri et al., 2021). The researcher took notes and asked for clarifications when needed, then collected the patient’s overall impression of the survey. In Belgium, two onco-coaches^1^ and one clinician provided further feedback based on the clinical perspective.

### 2.4. Data processing and statistical analysis

Descriptive analyses were carried out on patients’ demographic variables, health literacy, health LoC, and health related quality of life (all values are displayed in Table 2). Correlations and associations between measures were also calculated. The choice data resulting from the DCE were used to estimate the impact of the attribute levels on the respondents’ choices for treatment alternatives. A pooled dataset combining data from Italy and Belgium was used for these analyses because country of residence did not contribute to preference heterogeneity. Panel Latent Class models (LCM) were estimated to adjust for the multilevel structure of the data and to account for preference heterogeneity (Felli et al., 2009; Hensher et al., 2015). In this model, 3 attributes that are 5-year survival, chance of long-lasting skin problems and chance of extreme tiredness were considered to be linear, and 2 attributes, mode of administration (how the treatment is given to you) and severity of hair loss were considered to be non-linear and were therefore recoded using effects codes (Bech and Gyrd-Hansen, 2005). Based on model fit tests (AIC, Log likelihood), the model most suitable for our data was selected (models ranging from one to five classes were tested). The final utility equation is shown below. Results were considered statistically significant if *p* < 0.05.

**Table 2:** Socio-demographic, clinical, and psychological variables.

		**Italy**		**Belgium**	
		*Mean*	*St.Dev*	*Mean*	*St.Dev*
Age at survey completion		65.4	10	65.4	8.8
Age at diagnosis		63.9	10.3	63.5	9
		*Frequency*	*Percent*	*Frequency*	*Percent*
Gender	Male	88	55.7	89	59.7
	Female	70	44.3	60	40.3
Education	Compulsory school	0	0	6	4
	Secondary school	12	7,6	6	4
	University degree	37	23.4	30	20.1
Family & relationships	Single no kids	15	9.5	18	12.1
	Single with kids	12	7.6	17	11.4
	Partner with kids	64	40.5	38	25.5
	Partner no kids	67	42.4	76	51
Family history of cancer	Yes	45	28.5	37	24.8
	No	99	62.7	97	65.1
	Don’t know	14	8.9	15	10.1
Cancer stage	I, II	78	49.4	65	43.6
	III, IV	80	50.6	84	56.4
Type of treatment (more than one answer allowed)	No treatments	21	13.3	0	0
	Surgery	94	59.5	78	52.3
	Chemotherapy	55	34.8	88	59.1
	Immunotherapy	35	22.2	78	52.3
	Radiotherapy	35	22.2	46	30.9
	Other	18	11.4	12	8.1
	Don’t know	3	1.9	0	0
Lines of treatment	No treatment	72	45.6	72	48.3
	1 treatment	34	21.5	14	9.4
	2 treatments	14	8.9	15	10.1
	3 treatments	17	10.8	48	32.2
	> 3 treatments	21	13.3	0	0
Objective Health literacy (Newest Vital Sign)	High possibility of limited literacy	7	4.4	12	8.1
	Medium possibility of limited literacy	40	25.3	32	21.5
	Adequate literacy	111	70.3	105	70.5
		*Mean*	*St.Dev*	*Mean*	*St.Dev*
Mean Subjective Health Literacy (Chew Brief Literacy Scale)		3.1	0.8	3.6	0.8
MHLC - C	Internality	3.1	1	3	1
	Chance	2.8	1.1	3.4	0.8
	Doctors	4.7	0.9	4.7	0.6
	Other people	3.5	1.2	3.3	1
		*Number reporting some problems*	*(%)*	*Number reporting some problems*	*(%)*
EQ-5D-5L	EQ5D Mobility	55	35	76	51
	EQ5D Selfcare	19	12	40	26.8
	EQ5D daily activities	75	47.5	92	61.7
	EQ5D Pain/discomfort	103	65.2	99	66.4
	EQ5D Anxiety/depression	88	55.7	74	49

V_rta|c_ = β1_|c_ Mode infusion at hospital _for 12 h rta|c_ + β2_|c_ Mode infusion at hospital _for 24 h rta|c_ + β3_|c_ 5-year survival _rta|c_ + β4_|c_ Probability of long-lasting skin problems _rta|c_ + β5_|c_ Probability of extreme tiredness _rta|c_ + β6_|c_ Hair loss _some loss rta|c_ + β7_|c_ Hair loss _no loss rta|c_.

The systematic utility component (V) describes the observable utility that participant “r” belonging to class “c” reported for alternative “a” in choice task “t.” The attribute level estimates of each attribute are represented by β1–β7. Interaction terms between the attributes were not included as they were identified as statistically insignificant.

In addition to the above-specified utility function, an LCM with class assignment was fitted. Several demographic and disease related measures were tested for a significant contribution to the class assignment model (i.e., country, age, gender, health literacy, educational level, cancer stage, lines of treatment, having children and experience with types of treatment). Demographic, clinical and psychological variables were entered in the LCA model as dichotomous variables. Continuous variables including age and objective and subjective health literacy were dichotomized using a median split. With regards to health LoC, for each of the four dimensions of the multidimensional tool, a median split of the score was used to classify participants into low or high level of health LoC. For all 4 dimensions, patients were classified as either being among the low (representing lower internal/chance/doctors/others LoC) or high (representing higher internal/chance/doctors/others LoC) end of the spectrum. A significant coefficient in the class assignment model indicates that the attribute level contributes to the class assignment, while the sign of the coefficient reflects whether the impact is positive or negative.

Relative importance scores were calculated by computing the difference between the highest and the lowest estimates of the attribute level within each attribute. A value of 1 was given to the highest difference value, representing the attribute that was deemed most important by respondents. To calculate the relative distance between the most important attributes and all other attributes, the other distance values were divided by the largest difference values. These relative importance scores were calculated separately for each class, and then weighted according to class assignment probability to derive class-adjusted averages.

Finally, maximum acceptable risk (MAR) and minimum acceptable benefit (MAB) were calculated following Eqs. (1) and (2), respectively. The MAR is interpreted as the highest probability of adverse side effects that respondents are willing to accept a one-percentage point increase of the chance in treatment efficacy (and thus 5-year survival in our design), while the MAB is the minimum increase in effectiveness of the treatment that respondents would require to accept changes to a less desirable level in another attribute (e.g., from some hair loss to complete hair loss).


(1) $$MAR=\frac{\left(Q_{5yearsurvival}\right)}{\left(Q_{k=risk}\right)}$$

(2) $$MAB=\frac{\left(Q_{bestlevel}-Q_{worstlevel}\right)}{\left(Q_{5yearsurvival}\right)}$$

## 3. Results

A total of 560 cancer patients at different stages of the disease were approached across Italy and Belgium. Of these, 159 refused to participate and 94 initially gave their consent but later withdrew from the study, leaving 307 NSCLC patients in the final sample (*N* = 158 in Italy and *N* = 149 in Belgium).

Regarding the Italian patients who refused to complete the survey or dropped out, their mean age was about 70 years (SD = 9), with a slightly higher percentage of men than women (about 54 vs. 46% respectively). 54% were patients at cancer stage I-II, and 46% patients at stage III-IV. All patients at stage I-II underwent surgery, since surgery is the main treatment option for early-stage NSCLC, and among them only 13.15% underwent an adjuvant chemo or radiotherapy. Whereas patients at cancer stage III-IV underwent one or more lines of treatments. Their characteristics appeared not to differ a lot from patients that were enrolled and therefore agreed to participate (see Table 2).

The main reported reasons for refusing to participate or to drop out the study were: “I cannot make it (unspecified), I do not have time” about 32.4%; “I do not feel enough good psychologically and/or physically to participate” about 18.3% patients; “I am too old […] I am unable to fill out the survey on pc […] I do not have anyone who can help me” about 14.1%; “I do not have a suitable pc, I do not use pc” about 16.9% patients; 15.5% patients passed away in the following weeks after they have been contacted to participate.

Concerning patients from Belgium who refused or dropped out, we have less information available. 42.0% were patients at cancer stage I-II, and 48% patients at stage III-IV. Their main reported reasons were (percentages not available): “I do not have time”; “I do not feel enough good psychologically and/or physically to participate”; “I am unable to fill out the survey on pc (because no pc available or not able to work with it)”; language barrier.

### 3.1. Socio-demographic, clinical and psychological variables of enrolled patients

Table 2 shows socio-demographic, clinical and psychological data for patients in the two countries. Respondents did not differ significantly between the two countries in terms of age [t (305) = 0.003, *p* = 0.998)], age at diagnosis [t (303.469) = 0.288, *p* = 0.774)], gender [χ^2^ (1) = 0.511, *p* = 0.475], or family history of cancer [χ^2^ (2) = 0.572, *p* = 0.751]. The model comparing family and relationship status across the two countries was significant [χ^2^ (3) = 8.1, *p* = 0.045], however the standardized residuals indicated no significant differences between observed and expected frequency. The same was true for education [χ^2^ (2) = 7.248, *p* = 0.027], where expected and observed residuals did not differ significantly across Italian and Belgian participants. Participants were recruited across different stages of cancer: the final sample included 143 patients in stage I and II, and 164 in stage III and IV. The proportion of patients in different stages was matched across the two countries [χ^2^.(1) = 1.016, *p* = 0.313]. On average, patients in stage I and II were older (M = 67, SD = 9.2) than patients in stage III and IV [M = 64, SD = 9.4; t (305) = 2.7, *p* = 0.006].

*Health literacy* of respondents was not significantly different between Italy and Belgium when measured with an objective scale [χ^2^ (2) = 2.109, *p* = 0.348], while subjective/reported health literacy was significantly lower in the Italian sample compared to the Belgian sample [t (305) = −6.591, *p* < 0.001]. Participants’ age was negatively correlated with both objective (r = −0.180) and self-reported (r = −0.147) health literacy.

With regard to *health LoC*, Italian and Belgian participants did not differ in their tendency to believe that health is mainly their own responsibility [i.e., internality subscale; t (305) = 0.766, *p* = 0.444], in their beliefs about the degree of control that doctors have over health outcomes [i.e., doctors subscale; t (305) = −0.665, *p* = 0.506], or that significant others have over their health outcomes [i.e., others subscale; t (305) = 1.460, *p* = 0.145]. In contrast, Belgian participants were more likely to attribute their health outcomes to chance [i.e., chance subscale; t (204.412) = −5.985, *p* < 0.001]. Descriptive statistics for the four subscales are provided in Table 2.

Differences between Italy and Belgium in *health-related quality of life* were observed in participants’ mobility, selfcare, and daily activities, with Belgian patients deviating from level 1 (no problems at all) more frequently than Italian patients, and anxiety/depression with Italian patients deviating from level 1 more frequently than Belgian patients. Descriptive statistics for the subscales are reported in Table 2.

### 3.2. Preference elicitation

A two-class latent class model presented best model fit (Table 3). The average class probability for class 1 and 2 was 65 and 34%, respectively. In both classes, patients preferred treatments with a higher 5-year survival, lower probability of long-lasting skin problems and a lower probability of extreme tiredness. In both classes patients also preferred some hair loss and no hair loss over complete loss of hair. Furthermore, only in class 1 patients significantly preferred oral treatment over infusions. Dominant decision-making (i.e., non- trading between attributes) was relatively high with 66.5% of Italian respondents and 57% of Belgian respondents always choosing the option with the highest survival level.

**Table 3:** Preferences of lung cancer patients for treatment options based on the latent class analysis.

Attributes		Class1			Class2		
		Beta	SE	95% CI	Beta	SE	95% CI
How the treatment is given to you	Pill (ref)	0			0		
	Infusion in hospital 12 hours	−0.64***	0.13	−0.90; −0.38	0.04	0.12	−0.20; 0.28
	Infusion in hospital 24 hours	−0.47***	0.14	−0.74; −0.19	−0.17	0.13	−0.42; 0.07
Chance of surviving 5 years after starting this cancer treatment		0.44***	0.04	0.37; 0.51	0.07***	0.01	0.05; 0.09
Chance of long-lasting skin problems		−0.04***	0.01	−0.05; −0.03	−0.02***	0.00	−0.03; −0.02
Chance of being extremely tired		−0.06***	0.01	−0.07; −0.05	−0.02***	0.00	-0.03; −0.01
Severity of hair loss	Complete loss of hair (ref)	0			0		
	Some hair loss	0.93***	0.17	0.60; 1.26	0.63***	0.12	0.39; 0.87
	No hair loss	1.49***	0.20	1.10; 1.88	0.54***	0.13	0.30; 0.79
Mean class probability		0.66			0.34		
**Class assignment model**							
Constant		0.29	0.39	−0.48; 1.05			
High health literacy		1.00***	0.36	0.30; 1.69			
High educational level		1.24**	0.59	0.09; 2.39			
Elderly (aged >65 years)		−0.76**	0.34	−1.41; −0.10			
**Model fit measures**							
Loglikelihood		−1437.03					
AIC		2910.1					
Pseudo *R*^2^		0.44					

Beta values with ***, ** are significant at 1%, 5% respectively. Probability attributes are based on a one-point increase in probability. SE = standard error of the mean, CI = confidence interval, AIC = Akaike information criteria.

The model fit significantly improved when including health literacy, educational level and age in the class assignment model. Patients with a higher objective health literacy (NVS), and patients with a higher educational level were more likely to belong to class 1 while elderly patients (over 65 years) were less likely to belong to class 1. Variables such as gender, country, educational level, stage of disease and lines of treatment were not significant predictors of class membership.

**Figure 2:**
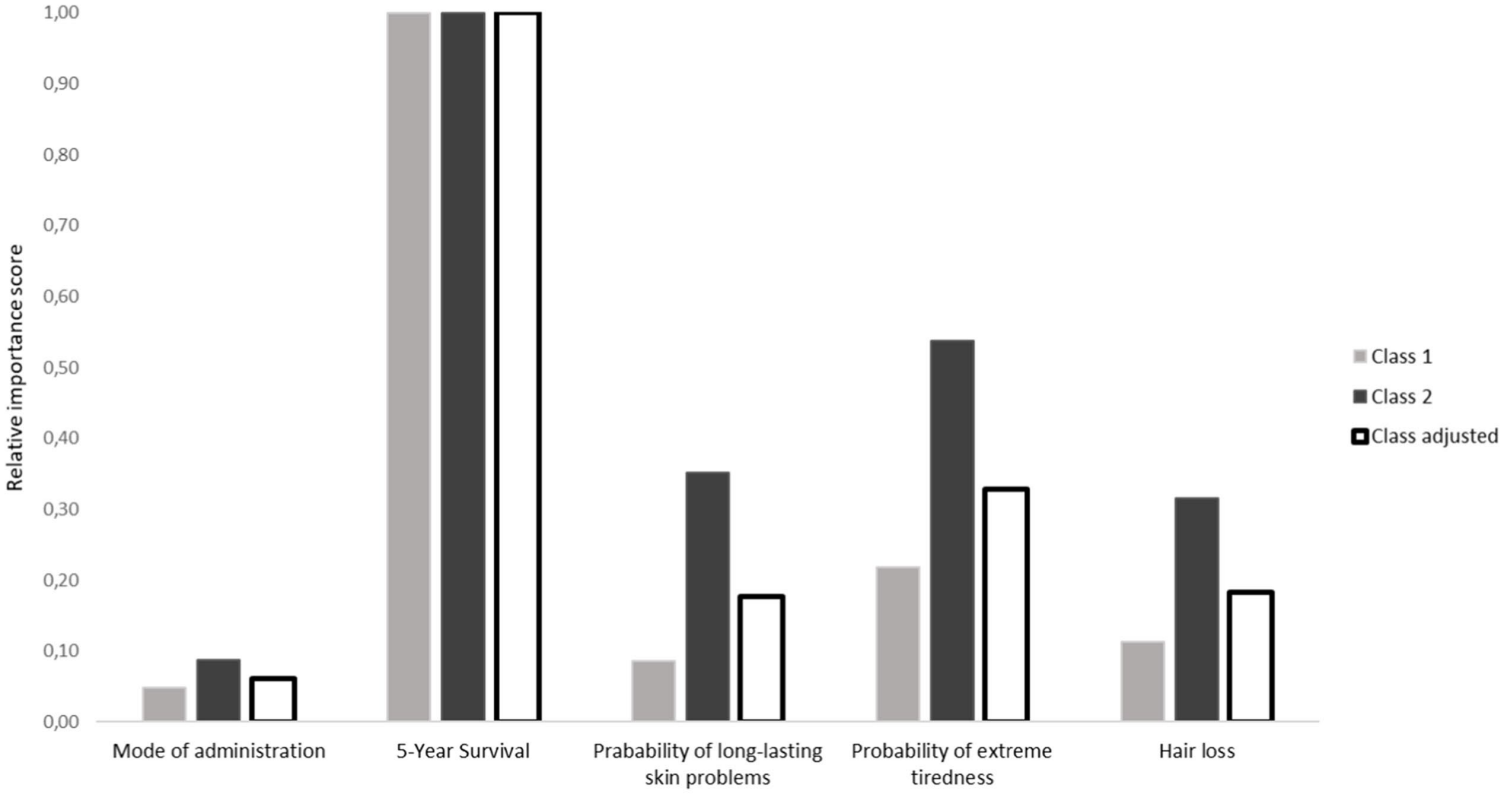
Relative importance score of attributes stratified per class of the LCA model as well as the class adjusted scores.

The relative importance scores were calculated separately for the two classes of the LCA, and adjusted for class probability (Figure 2). Values indicated that 5-year survival was most important in both classes, but the other attributes were relatively more important in class 2 compared to class 1 (Figure 2). This suggests that people who are more likely to belong to class 1 attach less importance to the treatment side effects relative to survival. The mode of administration was almost equally important for patients in class 1 and 2. In the two classes, all the attributes, including the side effects, and the mode of administration, contributed to patients’ decision-making because all these attributes were significant, but to a much smaller degree than survival. However, patients in class 2 (who are more likely to be older, less educated and less literate) placed more value on the side effects of treatment compared to those in class 1.

MAR and MAB outcomes can be found in Table 4. Adjusted for class probability, patients were willing to accept an 8.6 percentage point increase in probability of long-lasting skin problems for a 1 percentage point increase in 5-year survival. Likewise, they were willing to accept a 6.1 percentage point increase in probability of extreme tiredness for a 1 percentage point increase in 5-year survival. The minimum benefit required to accept a switch from taking a pill to an infusion at the hospital for 24 h was 1.6 percent point increase in 5-year survival, and 5.0 percent point increase in 5-year survival was required to accept switching from no hair loss to a treatment profile that would result in complete hair loss. Overall, these indexes are indicative of a strong acceptance of treatment related risks in exchange for an increased probability of survival. Large differences were found in the MAR and MAB between the two classes of the LCA model. In Class 1, the MAR for long lasting skin problems and extreme tiredness were, respectively, about 4 times and 2.5 times larger than in class 2, suggesting a higher risk tolerance in exchange for an increased survival (see Table 4). In line with this, MAB values were smaller in class 1 compared to class 2, indicating that a smaller benefit was needed in this class to accept the treatment side effects.

**Table 4:** MAR and MAB for a one percentage point change in chance of 5-year survival values based on the LCA output for the entire population and excluding non-traders.

	Entire population						Excluding non-traders					
	Class 1		Class 2		Class adjusted		Class 1		Class 2		Class adjusted	
	Mean (SE)	95% CI	Mean (SE)	95% CI	Mean (SE)	95% CI	Mean (SE)	95% CI	Mean (SE)	95% CI	Mean (SE)	95% CI
**MAR**												
Chance of long-lasting skin problems	11.6 (1.44)	8.80:14.47	2.8 (0.56)	1.75:3.95	**8.6 (0.94)**	6.78:10.48	4.3 (1.00)	2.35:6.28	1.6 (0.28)	1.05:2.18	**2.7 (0.45)**	1.84:3.62
Chance of being extremely tired	7.6 (0.59)	6.49:8.81	3.1 (0.48)	2.17:4.04	**6.1 (0.38)**	5.35;6.84	2.1 (0.22)	1.71:2.58	3.9 (1.18)	1.62:6.23)	**3.1 (0.66)**	1.80:4.49
**MAB**												
How the treatment is given to you	1.1 (0.32)	0.43:1.68	2.6 (1.83)	−0.96:6.19	1.6 (0.61)	0.38:2.79	0.7 (1.47)	−2.20:3.58	4.0 (2.95)	−1.75:9.81	2.6 (1.62)	−0.52:5.82
Severity of hair loss	3.4 (0.42)	2.54:4.21	8.1 (2.48)	3.27:13.00	**5.0 (0.84)**	3.37:6.64	1.9 (2.27)	−2.53:6.41	16.6 (4.37)	8.02:25.15	**10.5 (2.60)**	5.42:15.60

In bold are reported the values of MAR and MAB that were affected by significant changes by considering the entire sample (left column) vs. traders only (right column), for each referred attribute.

A sensitivity analysis was done for the MAR and MAB values excluding non-trading respondents (Table 4). MAR for skin problems and tiredness and MAB for hair loss significantly differs between the entire and the trading population. The filtered sample, with a less outspoken preference for survival above all other attributes, was on average (i.e., class adjusted) less willing to accept the side effects of a treatment for a 1% increased probability of surviving 5 years from the diagnosis, with a reduction of 5.8 percent points in the willingness to accept long-lasting skin problems and a reduction of 2.8 percent points in the acceptance of extreme tiredness. The same pattern of results was seen in the minimum benefit required to accept the worst side effects of treatment, where patients required a 2.7 percent point increase in the probability of 5-year survival in order to accept a switch in the mode of administration (from taking a pill at home to having to stay in the hospital for 24 h to get an infusion), and 10.6 percent point increase in 5 year survival to accept a complete loss of hair. Differences across the two classes mirrored those observed in the entire population, with the exception of the MAR for extreme tiredness which went in the opposite direction (i.e., higher risk acceptance in class 2 vs. class 1).

A separate LCA model was fitted to investigate the influence of health LoC on preference heterogeneity (Table 5). The lack of convergence in the model was the reason for fitting a separate LCA model for the LoC. The three significant predictors from the previous LCA (i.e., health literacy, education and age) were entered in this model alongside LoC. We found that two sub-scales of LoC, i.e., high internal and high chance, predicted class membership in a similar fashion: patients with high internal and high chance LoC were less likely to belong to class 1. In line with the previous model, patients with a high level of health literacy, and those with a high educational level were more likely to belong to class 1 while elderly patients were less likely to belong to class 1.

**Table 5:** Preferences of lung cancer patients for treatment options based on the latent class analysis with health locus of control.

**Attributes**		**Class1**			**Class2**		
		**Beta**	**SE**	**95% CI**	**Beta**	**SE**	**95% CI**
How the treatment is given to you	Pill (ref)	0			0		
	Infusion in hospital 12 hours	−0.65***	0.13	−0.91; −0.39	0.05	0.12	−0.18; 0.28
	Infusion in hospital 24 hours	−0.44***	0.15	−0.72; −0.15	−0.18	0.13	−0.43; .07
Chance of surviving 5 years after starting this cancer treatment		0.44***	0.03	0.38; 0.51	0.07***	0.01	0.05; 0.08
Chance of long-lasting skin problems		−0.04***	0.01	−0.05; −0.03	−0.02***	0.00	−0.03; −0.01
Chance of being extremely tired		−0.06***	0.01	−0.07; −0.05	−0.02***	0.00	−0.03; −0.02
Severity of hair loss	Complete loss of hair (ref)	0			0		
	Some hair loss	0.92***	0.16	0.59; 1.25	0.63***	0.12	0.40; 0.86
	No hair loss	1.52***	0.19	1.14; 1.89	0.52***	0.12	0.28; 0.77
Mean class probability		0.655			0.345		
**Class assignment model**							
Constant		0.59	0.51	−0.42; 1.59			
Health locus of control	internal	−0.95***	0.35	−1.64; −0.27			
	chance	−0.79**	0.34	−1.45; −0.13			
	doctor	0.62*	0.35	−0.07; 1.31			
	others	0.63*	0.34	−0.05; 1.29			
High health literacy		1.00***	0.37	0.28; 1.72			
Elderly (aged >65 years)		−0.81**	0.35	−1.50; −0.12			
High education		1.34**	0.62	0.13; 2.55			
**Model fit measures**							
Loglikelihood		−1427.59					
AIC		2899.2					
Pseudo *R*^2^		0.44					

Beta values with ***, ** are significant at 1%, 5% respectively. Probability attributes are based on a one-point increase in probability. SE = standard error of the mean, CI = confidence interval, AIC = Akaike information criteria.

## 4. Discussion

Using DCE, this study set out to address two main clinical questions relative to patient preferences for lung cancer treatments. First, we assessed preferences for five treatment attributes in order to identify their relative importance and quantify the maximum acceptable risk (MAR) and the minimum acceptable benefit (MAB) that patients would accept for treatment alternatives. Next, we explored the clinical, demographic and psychological variables that may explain preference heterogeneity. Respondents indicated that the probability of being alive 5 years from the start of cancer treatment was the most important attribute in their treatment decision. Overall, patients were willing to accept treatment related risks to increase their probability of survival. Importantly, this study revealed that age, educational level, objective health literacy and health LoC explained preference heterogeneity.

The strong preference for 5-year survival is consistent with previous literature on preferences of cancer patients (Veldwijk et al., 2019; Collacott et al., 2021). The difference attributed to survival relative to other attributes in our study is particularly striking. Over 50% of the respondents in this study exhibited dominant decision-making strategies in which they always chose the option that had the highest chance of survival. This dominant decision-making could be explained by the rapid tumor progression and the high mortality rate in lung cancer compared to other cancers (Siegel et al., 2021). While this effect could also reflect a strategy to reduce the intellectual burden of the task (Gilovich et al., 2002; Hauser, 2014), the deadly nature of lung cancer raises the possibility that non-trading behavior may reflect true extreme preferences for survival over other attributes (Hess et al., 2010). A recently published study showed that proximity to death strongly influences the marginal rates of substitution between survival improvements and risks of adverse events (Marsh and Krucien, 2022). However, other studies of NSCLC patient preferences have found no difference in the prioritization of progression-free survival (i.e., time without tumor progression) over improving tumor- associated symptoms (i.e., shortness of breath, pain, coughing; Mühlbacher and Bethge (2015). It is possible that the way the survival attribute was worded may have influenced patients’ subjective and emotional reaction to this attribute. In our study we defined survival as the “chance of surviving 5 years after starting this cancer treatment.” This definition focuses on the benefit that patients may derive in terms of increased life expectancy without mentioning the side effects of treatment and anchoring the gain in time. In contrast, other preference studies with lung cancer patients have characterized survival as “progression-free survival” or “progression- free survival with side effects” (Mühlbacher and Bethge, 2015; Janssen et al., 2020). Our definition was not as clear regarding the QoL during this period so patients may have assumed that 5-year survival meant symptom free, and 5 years is extremely long survival compared to what lung cancer patients may realistically expect. Conflicting evidence about the attribute “survival” may be also related to the difficult understandability of the concept “progression free survival” by patients. Different interpretations of the attributes may influence preferences.

MAR and MAB values revealed class differences in benefit–risk trade-offs. Specifically, respondents who were more likely to belong to the class which assigned less importance to treatment side effects (class 1) tended to accept a higher probability of risks in exchange for an increased survival and required a smaller benefit to switch to a less favorable frequency of side effects. Nevertheless, similar MAR patterns were found across the two LCA classes. Overall, these results highlight the importance of considering patient characteristics to improve communication between oncologists and patients regarding treatment options. By better understanding who the patient is, care providers can better provide patients with the information that they need in order to make an informed treatment decision. A greater involvement of patients in medical decision-making may in turn improve adherence to treatment (Jimmy and Jose, 2011; Kardas et al., 2013; Janssens et al., 2021).

Among several demographic and psychological variables which are known to impact patient preferences for health-related decisions, age, health LoC, educational level and health literacy were found to be significant predictors. First, elderly patients were more likely to belong to the class which attaches greater importance to the treatment side effects. This effect could be the result of a negative attitude towards the side effects developed through repeated experience with cancer treatment in the past. Patients aged 65 or above are more likely to be in an advanced stage of the disease with lower life expectancy, or on average have experience with more different treatment lines, and consequently they might be more concerned or focused on their quality of life compared to younger respondents. The mode of administration was the least important attribute for older patients, although it was still significant. Although research has shown that home therapy is an acceptable and safe alternative to hospital treatment for patients and may improve compliance and satisfaction with treatment (Borras et al., 2001) it is less important because it has a lower impact on older patients’ life style (no kids/work/caregiving etc.) and quality of life as compared to fatigue, nausea, or skin rash. People with high internal LoC and people with high chance LoC (external LoC) were also likely to belong to the class which assigns greater importance to the treatment side effects. It is possible that feeling able to control what happens around one’s health (i.e., high internal LoC) and thinking that health outcomes are only attributable to chance (i.e., high chance LoC) results in similar preferences in the case of cancer therapies. The former might feel high responsibility for the side effects, and perceive that, in case of a worse impact of therapies on their own quality of life, the distress may be too much to bear. The latter might feel concerned about the perceived lack of control over the side effects – which may in turn generate uncontrolled anxiety. Both cancer patients with internal and external LoC tend to choose therapies with limited side effects, since they both do not feel enough confidence in managing the impact of therapies on their quality of life. Also, they both may be convinced that others, for example health care workers, are the only ones able to handle cancer therapies’ side effects. Indeed, Gibek and Sacha (2019) found that cancer patients tend to believe that they have a moderate impact on their own health status in general, independently of their LoC profiles, as compared to those in the general population.

With regards to educational level and health literacy, our findings suggest that people with higher education and higher health literacy were more likely to attribute less importance to the treatment side effects. In a 2015 study of cancer patients’ preferences, Mühlbacher and Bethge (2015) found that educational level explained preference heterogeneity. Patients with higher education and literacy might also better understand the definitions of the attributes – so that they are not daunted by medical terms included in the explanations of the side effects - or other variables not explored in the current study might explain the lesser importance attributed by this class of patients to treatment side effects. We hypothesize that this might be the result of a greater trust in science and in scientific practices among highly educated people, or people who have had extensive experience with illness. Future research could test this hypothesis by exploring correlations between the degree to which respondents trust medical procedures and their level of education and health literacy. From the perspective of clinical experience, we expected to find that patients at more advanced stages for whom the life expectancy is relatively short might be more focused on survival compared to patients at earlier stages where surgical intervention is still a viable option. Contrary to our expectations, however, we did not observe any effects associated with cancer stage on preferences, revealing that in the face of a “deadly cancer” diagnosis all patients have survival as a priority above all other aspects, independent of how bad their prognosis actually might be.

The primary strength of this study is that we followed a rigorous methodological approach, including an extensive qualitative phase which informed the selection of attributes (Durosini et al., 2021; Janssens et al., 2021; Petrocchi et al., 2021). Rather than opting for a literature-based approach, focus groups were used to collect patients’ perspective and identify relevant attributes. Moreover, the multi-centric and multi-country nature of the research increases the generalizability of the results. Among several important advantages a web-based survey allowed us to include interactive educational videos, which provided instructions in plain language and offered another type of learning modality for participants (Oliveri et al., 2021). This study also had some limitations. First, there was a high number of respondents who displayed dominant decision-making behavior, and consequently the MAR and MAB values might not be truly reflective of the patients’ benefit–risk trade off. Although non-trading behavior is present in all choice experiments to some extent, in this study the proportion of respondents who systematically chose survival was particularly high. In order to account for this, we conducted a sensitivity analysis and presented the results both with and without these non-traders to show how they impact the preferences found. A further limitation is that the sample comprised relatively older and vulnerable patients, therefore caution must be applied when generalizing to other cancer populations which may be comprised of younger individuals. Moreover, due to the SARS-CoV-2 pandemic, the original research protocol had to be adapted to a fully remote survey. While this had several advantages, including the possibility of reaching a wider and geographically distant population in a shorter timeframe and a reduced burden on the patients, it also carried some challenges which should be considered when conducting web-based preference studies (Oliveri et al., 2021). In particular, dwelling on the number of patients who refused to participate in the study or dropped out, we notice that one-third from the Italian population were elder patients who struggled in using new technologies to complete the survey (a motivation also reported by Belgian patient population). There is a possibility that enrolled patients who were helped by their families and careers during the completion of the questionnaire were influenced in their interpretation of the attributes. If so, patients’ preferences measured in this study might not fully reflect their true perspectives. Future studies using web- based modes of administration of DCE should strive to minimize this risk by stressing the importance of allowing assistance for resolving technical issues only.

## 5. Conclusion

Our study confirms a preference for survival over other attributes among cancer patients, independent of the cancer stage or the experience with the disease. Non-trading behavior is present in all choice experiments to some extent, however in this study the proportion of respondents who systematically focused on survival was particularly high. Although this effect could reflect a strategy to reduce the intellectual burden of the task and attention must also be paid to the wording of the attributes, the deadly nature of lung cancer suggests that non-trading behavior may reflect true extreme preferences for survival over other attributes. Future research is needed to further characterize this effect. Age, objective health literacy and locus of control accounted for heterogeneity in patients’ preferences for cancer therapies. These aspects can guide regulators and other stakeholders on adapting the therapeutic protocols on the basis of patients’ conditions and socio-demographic parameters. Results can also be helpful to the industry to inform the Development Phase for other NSCLC assets (i.e., 1. To select prioritized targets; 2. To set priorities for studies; 3. To prioritize assets to progress into Phase 3). As well as evaluating results across countries provides insights into the reliability of preferences for informing decisions. These results may contribute to regulatory guidance on the use of preferences methods in decision making. In the post-marketing context, findings may potentially be used to update the clinical section of the label and to inform the patient label.

## Data availability statement

The datasets generated for this study will not be made publicly available. Participants did not provide consent for the sharing of survey answers with parties other than the researchers. Further inquiries can be directed to the corresponding author/s. The raw data supporting the conclusions of this article will be made available by the authors under appropriate request verification.

## Ethics statement

The studies involving human participants were reviewed and approved by Ethical Committee of the European Institute of Oncology IRCCS (IEO, Milan, Italy; reference R1142/20-IEO 1206) and the “Ethische Commissie Onderzoek UZ/KU Leuven” (Belgium; reference S63007). The patients/participants provided their written informed consent to participate in this study.

## Author contributions

SO, JV, GW, RJ, MYS, IS, IH, and GP designed the research and the experiment. Recruitment was organized and performed by SO, LL, SP, RJ, ES, KN, MV, EL, HeD, PD, HaD, FP, MC, GG, MG, and IH. Data were collected and database completed by SO, LL, JV, SP, and RJ. The analysis was conducted by JV, LL, SO, SP. SO, LL, and JV produced the first draft of the manuscript, which was subsequently revised and finalized by all authors. All authors contributed to the article and approved the submitted version.

## Funding

The Patient Preferences in Benefit–Risk Assessments during the Drug Life Cycle (PREFER) project has received funding from the Innovative Medicines Initiative 2 Joint Undertaking under grant agreement No 115966. This Joint Undertaking receives support from the European Union’s Horizon 2020 research and innovation program and EFPIA. This text and its contents reflect the PREFER project’s view and not the view of IMI, the European Union or EFPIA.

## Conflict of interest

Author MYS was employed by company Alexion Pharmaceuticals, Inc., Boston, MA, United States.

The remaining authors declare that the research was conducted in the absence of any commercial or financial relationships that could be construed as a potential conflict of interest.

## Publisher’s note

All claims expressed in this article are solely those of the authors and do not necessarily represent those of their affiliated organizations, or those of the publisher, the editors and the reviewers. Any product that may be evaluated in this article, or claim that may be made by its manufacturer, is not guaranteed or endorsed by the publisher.

## Author disclaimer

This text and its contents reflect the PREFER project’s view and not the view of IMI, the European Union or EFPIA.

## Footnotes

^1^Cancer coaching is a person-centered care process which promotes self-determined behaviour change through a supportive and collaborative partnership. Cancer Coaches are trained volunteers who provide comfort and practical information for patients, and support patients as they work toward health-related goals.
